# Analysis of Antidepressant-like Effects and Action Mechanisms of GSB-106, a Small Molecule, Affecting the TrkB Signaling

**DOI:** 10.3390/ijms222413381

**Published:** 2021-12-13

**Authors:** Yulia V. Vakhitova, Tatiana S. Kalinina, Liana F. Zainullina, Anastasiya Yu. Lusta, Anna V. Volkova, Nikita V. Kudryashov, Tatiana A. Gudasheva, Alexander A. Shimshirt, Ilya A. Kadnikov, Mikhail V. Voronin, Sergei B. Seredenin

**Affiliations:** 1 Department of Pharmacogenetics, FSBI “Zakusov Institute of Pharmacology”, Baltiyskaya Street 8, 125315 Moscow, Russia; zainullinalf@gmail.com (L.F.Z.); Lusta.Anastasiia17@mail.ru (A.Y.L.); ikadnikov@gmail.com (I.A.K.); dnamed@mail.ru (M.V.V.); seredeninpharm@mail.ru (S.B.S.); 2 Department of Psychopharmacology, FSBI “Zakusov Institute of Pharmacology”, Baltiyskaya Street 8, 125315 Moscow, Russia; volk16@inbox.ru (A.V.V.); kunvi@mail.ru (N.V.K.); spring05-79@yandex.ru (A.A.S.); 3 Department of Medicinal Chemistry, FSBI “Zakusov Institute of Pharmacology”, Baltiyskaya Street 8, 125315 Moscow, Russia; tata-sosnovka@mail.ru

**Keywords:** BDNF, dipeptide mimetics, TrkB signaling, antidepressant, unpredictable chronic mild stress

## Abstract

Induction of BDNF-TrkB signaling is associated with the action mechanisms of conventional and fast-acting antidepressants. GSB-106, developed as a small dimeric dipeptide mimetic of BDNF, was previously shown to produce antidepressant-like effects in the mouse Porsolt test, tail suspension test, Nomura water wheel test, in the chronic social defeat stress model and in the inflammation-induced model of depression. In the present study, we evaluated the effect of chronic per os administration of GSB-106 to Balb/c mice under unpredictable chronic mild stress (UCMS). It was observed for the first time that long term GSB-106 treatment (1 mg/kg, 26 days) during ongoing UCMS procedure ameliorated the depressive-like behaviors in mice as indicated by the Porsolt test. In addition, chronic per os administration of GSB-106 resulted in an increase in BDNF levels, which were found to be decreased in the prefrontal cortex and hippocampus of mice after UCMS. Furthermore, prolonged GSB-106 treatment was accompanied by an increase in the content of pTrkB^706/707^ in the prefrontal cortex and by a pronounced increase in the level of pTrkB^816^ in both studied brain structures of mice subjected to UCMS procedure. In summary, the present data show that chronic GSB-106 treatment produces an antidepressant-like effect in the unpredictable chronic mild stress model, which is likely to be associated with the regulation of the BDNF-TrkB signaling.

## 1. Introduction

Major depressive disorder, commonly referred to as clinical depression, is a severe mental chronic disease that has a high prevalence in almost all developed countries, with more than 280 million people affected worldwide (approximately 4.4% of the population), and is expected to become the leading cause of disease burden by 2030 [1]. Complicated etiology of depression involves large number of factors including stressful life events, genetic risk, and type of personality response to the adverse life experiences [2]. Multiple basic and clinical studies proposed that combination of those factors causes disturbances within mesocorticolimbic circuits (nucleus accumbens, prefrontal cortex and hippocampus), thus contributing to the pathophysiology and the core symptoms of depression [3]. Indeed, magnetic resonance imaging (MRI) revealed smaller volumes of hippocampus, the inferior anterior cingulate, and the orbital prefrontal cortex in patients suffering from depression that are consistent with the post-mortem studies and the data on animal models of depression-like behavior [4]. Lower volume of hippocampal and prefrontal cortex found in the subjects with depression are believed to be related to the neuronal atrophy through an increase in the apoptosis-associated DNA fragmentation and necrotic neuron death, as well as with alterations of synaptic reorganization proteins and, predominantly, with the reactive astrogliosis in the distinct hippocampal regions [5]. 

Although the neurobiology of depression is still not clearly understood, the monoamine-based, the glucocorticoid-dependent, the glutamate-driven, the neurotrophin signaling deficiency, the neuroinflammatory, the impaired brain circuit and networks hypotheses have been proposed [6,7,8,9,10]. To date, clinically active antidepressants target primarily the monoamine systems in the brain, inhibiting the reuptake of monoamines (selective serotonin or/and noradrenaline and dopamine reuptake inhibitors, tricyclic antidepressants), decreasing their degradation rate (inhibitors of monoamine oxidase), or acting on receptors (noradrenergic antagonist-specific serotonin antagonist, serotonin modulating antidepressants) [11]. However, the long onset time for antidepressants effects (several weeks to months), a significant number of patients that do not respond to typical antidepressants and a broad range of side effects significantly limit the efficacy of treatment [12]. Experimental studies have reported that the delayed onset of antidepressants action is associated with a slowly developed increase and rearrangements of synaptic strength in the depression-related circuits, requiring changes in the gene expression and protein translation that are dependent, to some extent, on the engagement of BDNF/TrkB pathways [13,14]. Numerous basic research findings confirm that typical antidepressants increase the BDNF mRNA and protein expression as well as the TrkB activation in the hippocampus and the cortex [3,7,15], thus promoting the structural plastic changes associated with the antidepressant action mechanisms [8,16,17]. Moreover, conventional antidepressants have been shown to rapidly increase the phosphorylation of Tyr^706/707^ at the TrkB catalytic domain and the phospholipase Cγ-binding site (Tyr^816^), while leaving the phosphorylation of the Shc-interaction site Tyr^515^ unaffected [18,19,20]. A rapidly developing and sustained antidepressant effect of ketamine which depends on the NMDA receptors inhibition, leads to the activation of mTORC1 complex followed by an increase in the translation and release of BDNF, an enhancement of BDNF/TrkB signaling, and a subsequent activation of the related cellular plasticity cascades [21]. Casarotto and colleagues have recently reported that structurally unrelated antidepressants (fluoxetine, imipramine, moclobemide, ketamine, esketamine, R,R-HNK) directly bind to transmembrane region of dimerized TrkB and potentiate the receptor activation by BDNF [22]. Overall, a number of previous observations as well as some more recent evidence emphasize the significance of TrkB as a valuable target for antidepressants [23,24] and reaffirm the critical role of BDNF/TrkB signaling in the pathophysiology of depression and in antidepressants action [7,25,26]. 

The given data indicate the importance of BDNF/TrkB pathway for depression, leading to several proposed approaches aiming to restore or enhance neurotrophin signaling, such as: a local delivery of neurotrophin by engineered cells or viral vectors; an increase of BDNF levels and the neurotrophin effects; a regulation of TrkB receptor synthesis and dimerization; an indirect Trk receptors activation via transactivation mechanisms; a modulation of individual steps in the desired signal transduction pathway; an interaction with TrkB receptor (e.g., small peptide and non-peptide compounds for TrkB); a functional mimicry [23,27,28,29]. Although the intracerebral administration of BDNF produced an antidepressant-like effect in animal models of depression [30,31], clinical use of neurotrophin is limited, mainly due to its suboptimal pharmacokinetic properties and side effects. One of the ways to overcome these factors is to augment BDNF/TrkB pathway with small molecules that could function as TrkB ligands or enhance neurotrophin signaling [27]. Several low-molecular-weight peptide and non-peptide compounds with favorable pharmacokinetic profiles acting on the BDNF/TrkB pathway and exerting antidepressant properties in various depression-like animal behavioral models have been developed to date. The best characterized and well-studied examples are the 7,8-dihydroxyflavone [32,33] and its synthetic derivative [34], the low-molecular weight TrkB antagonist with anxiolytic and antidepressant activity ANA-12 [35], and the low molecular weight BDNF mimetic GSB-106 [36].

**Figure 1:**
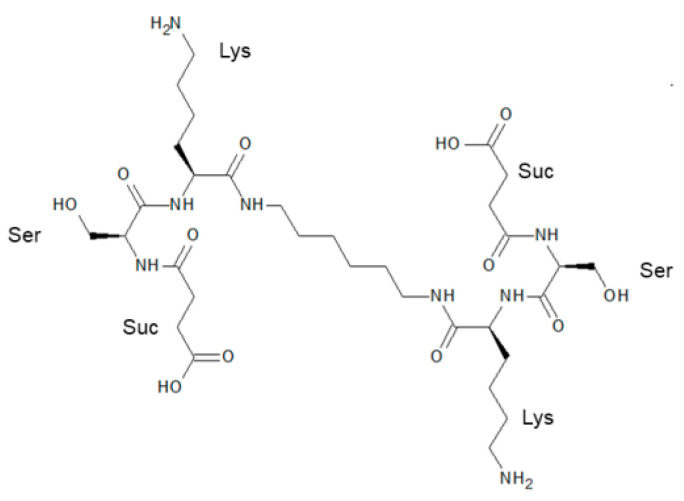
Chemical structure of GSB-106.

A crystallographic analysis, on par with mutagenesis studies and gain-of-function approaches based on chimeric recombination of BDNF protein, allowed to propose the amino acid residues, implicated in neurotrophin interaction with TrkB. In particular, residues 91–97 of the 4th loop of BDNF (Thr91-Met92-Asp93-Ser94-Lys95-Lys96-Arg97) have been recognized as one of the sites for neurotrophin/TrkB complex formation (reviewed in [37,38]). Orally available homodimeric dipeptide GSB-106 (bis-(*N*-monosuccinyl-*L*-seryl-*L*-lysine) hexamethylenediamide; Figure 1), which resembles the part of BDNF loop 4, has been recently developed at the Zakusov Institute of Pharmacology [36]. Namely, GSB-106 has been designed based on the BDNF loop 4 β-turn amino acid sequence -Asp93-Ser94-Lys95-Lys96-, where the putative TrkB-binding residues -Ser94-Lys95- were retained, the upstream residue -Asp93- was replaced by its bioisostere, a succinic acid residue, and -Lys96- was substituted by the amide group; C-terminal dimerization was performed using oligomethylenediamine spacer [36,39]. The compound has been shown to exert a trophic effect, up-regulate the TrkB phosphorylation and downstream PI3K/Akt, MAPK/Erk and PLCγ signaling pathways in vitro [40,41]. Furthermore, GSB-106 has been found to act as a partial TrkB receptor agonist, promoting protection of serum-deprived neuronal-like cells by counteracting cell apoptosis through the activation of TrkB-dependent pro-survival mechanisms, including inactivation of pro-apoptotic BAD protein and suppression of caspases 9 and 3/7 [42]. Behavioral studies revealed the substantial antidepressant-like activity of GSB-106. Particularly, GSB-106, administered to rodents either orally or intraperitoneally, under acute, subchronical or chronical conditions demonstrated an antidepressant-like activity in the Porsolt test, a tail suspension test and the Nomura water wheel test, as well as in the chronic social defeat stress model and in the inflammation-induced model of depression [36,43,44,45,46]. 

Generally, a panel of rodent tests and models is used to evaluate the potential antidepressant effect of the compounds. Based on its close resemblance to certain human depressive symptoms, unpredictable chronic mild stress (UCMS) is considered as one of the most translationally-relevant and valuable models for studying the pathophysiology of depression and the efficacy of antidepressants in animals [47]. It has been observed that rats and mice subjected to UCMS demonstrate anhedonia in a sucrose preference test and depression-like scores in the tests of learned helplessness such as the Porsolt test [48,49,50], while antidepressants eliminate those manifestations [51,52]. Most studies usually raise the question of drug efficacy in the settings of the developed behavioral abnormalities, and the course of potential antidepressants is initiated after several weeks of ongoing stress stimuli [53]. It has been widely shown that chronic stress-induced behavioral changes are associated with neuronal atrophy and reduced levels of BDNF and phosphorylated/activated TrkB in the prefrontal cortex and the hippocampus in rodents, while almost all antidepressants can restore BDNF level and activate TrkB receptors in rodent chronic stress models (reviewed in [7]). 

The purpose of this study is to examine the potential antidepressant-like effects of GSB-106 as demonstrated by an UCMS model of depression in Balb/c mice and to evaluate whether this effect is mediated through amelioration of BDNF/TrkB signaling.

## 2. Results

### 2.1. Effect of Chronic GSB-106 Administration on Depressive-like Responses in the Porsolt Test

**Figure 2:**
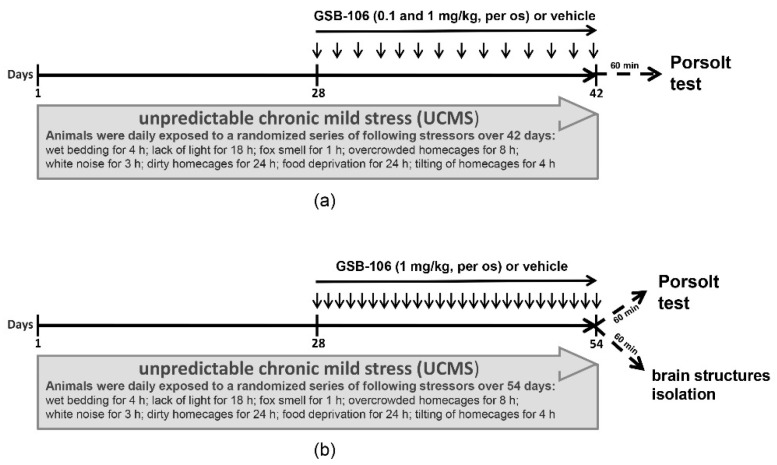
Schedule of unpredictable chronic mild stress experiment. Mice were exposed to UCMS for 28 days, after which, GSB-106 or vehicle were administered over (**a**) 14 days (14 sequential per os treatments, 1 time per day) or (**b**) 26 days (26 sequential per os treatments, 1 time per day) during the ongoing stress.

**Figure 3:**
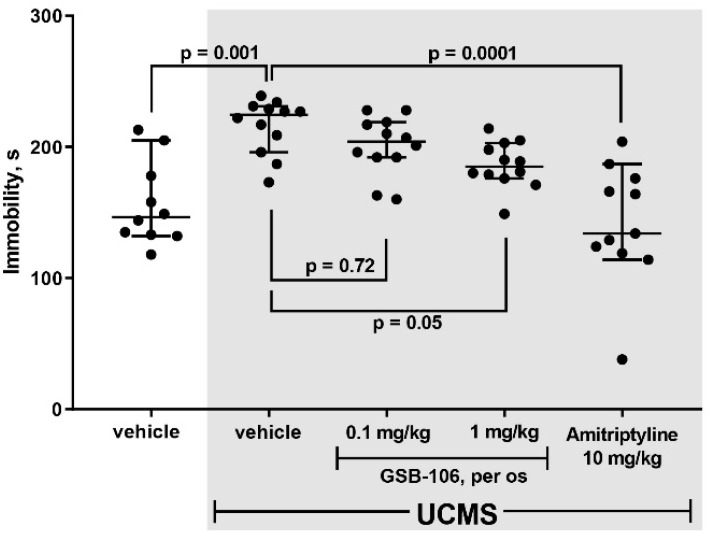
Effect of GSB-106 (0.1 or 1 mg/kg, 14 per os treatments) on the depressive-like behavior of mice in the Porsolt test. The results are presented as means ± SEM. The significance of intergroup differences was estimated by one-way analysis of the variance (ANOVA), followed by Tukey’s multiple comparisons test.

The GSB-106 compound administered to mice for 14 days in parallel with ongoing UCMS (Figure 2a) dose-dependently contributed to a decrease in the immobilization time in the behavioral test compared to the untreated animals (Figure 3). 

As shown in Figure 3, stressed mice exhibited a 1.4-fold increase (F = 12.83; *p* = 0.001) in immobilization time compared to non-stressed animals in the Porsolt test. A higher dose (1 mg/kg, per os) of GSB-106 attenuated depressive-like responses in mice by 1.2 (F = 12.83; *p* = 0.05) times compared to stressed mice treated with vehicle. Amitriptyline (10 mg/kg, per os) reduced the dysphoric behavior of stressed animals by 1.5 times (F = 12.83; *p* = 0.0001) compared to the control stressed mice. Thus, the preliminary stage of assessing the behavioral effects of substances in UCMS model indicated a dose-dependent anti-dysphoric effect of GSB-106. 

**Figure 4:**
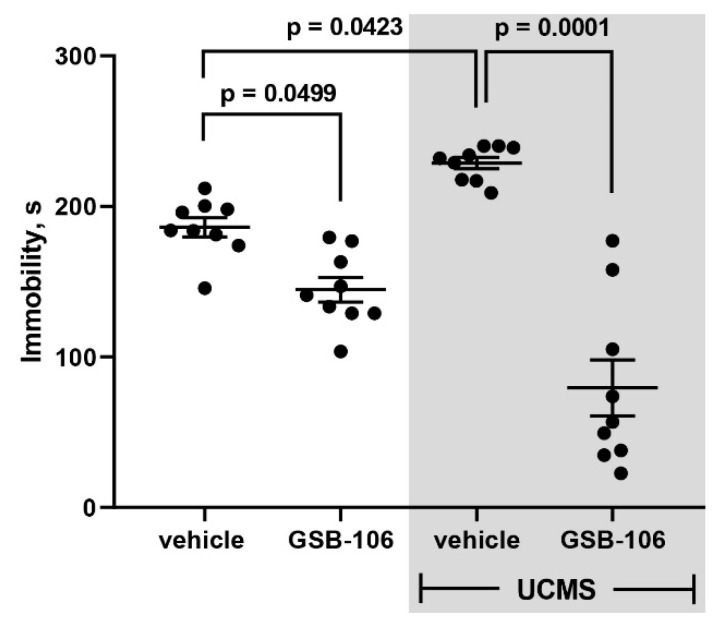
GSB-106 (1 mg/kg, 26 per os treatments) attenuates depressive-like behavior of mice in the Porsolt test. The results are presented as means ± SEM. The significance of intergroup differences was estimated by one-way analysis of the variance (ANOVA), followed by Tukey’s multiple comparisons test.

After the effective dose (1 mg/kg) was determined, antidepressant-like activity and underlying mechanisms of GSB-106 were evaluated upon the prolonged use of the compound (26 administrations versus 14) (Figure 2b). As follows from the results of the behavioral assessment GSB-106 effects in Porsolt test (Figure 4), GSB-106 caused a 1.3-fold decrease in the immobilization periods compared to the vehicle (F = 34.58; *p* = 0.0499) in the absence of UCMS. In the vehicle-treated mice group subjected to UCMS a 1.2 folds increase in immobilization duration was registered (F = 34.58; *p* = 0.0423) compared to non-stressed animals treated with vehicle. In mice exposed to UCMS, chronic GSB-106 treatments decreased the immobilization periods by 2.9 folds (F = 34.58; *p* = 0.0001), compared to the stressed mice treated with vehicle. Thus, the Porsolt test data indicate the anti-dysphoric effect of GSB-106 after 26 days of oral administration in Balb/c mice. At the same time, animals subjected to the stress procedure exhibited a deeper antidepressant effect of the substance compared to the corresponding control. 

### 2.2. Effects of Chronic GSB-106 Administration on BDNF Content and TrkB Site-Specific Phosphorylation in the Prefrontal Cortex and the Hippocampus of Mice 

**Figure 5:**
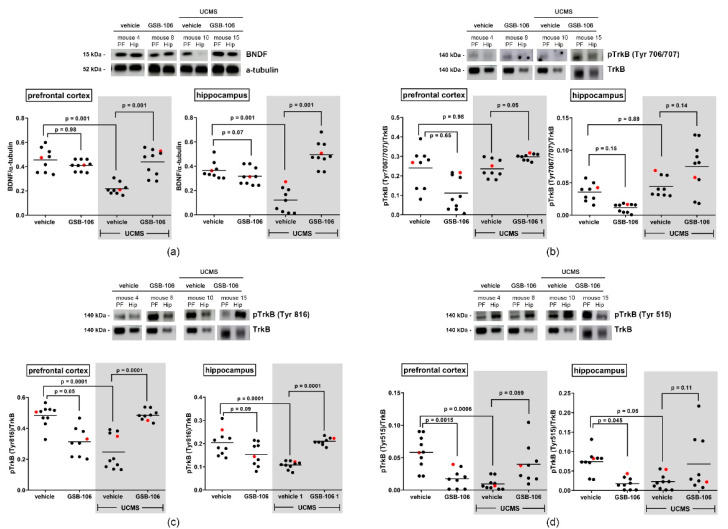
The effect of GSB-106 on the BDNF content and the site-specific phosphorylation of TrkB receptor in the prefrontal cortex and the hippocampus of mice subjected to UCMS. (**a**) BDNF, (**b**) pTrkB(Tyr^706/707^), (**c**) pTrkB(Tyr^816^), (**d**) pTrkB(Tyr^515^) proteins expression in mice prefrontal cortex and hippocampus. After the completion of UCMS procedure and the compound treatment, mice were decapitated and brain structures were collected. Protein extracts were subjected to polyacrylamide gel electrophoresis and transferred for Western blotting. Blots were probed with anti-BDNF, anti-phosphorylated TrkB antibodies and then reprobed with anti-TrkB antibody, anti-a-tubulin antibodies. For every picture, representative images for immunoblots are shown on the top panels, and quantitative data are shown on the bottom panels (red dots on the graphs correspond to mice 4, 8, 10 and 15; dark dots are all mice samples in the group). All data are expressed as mean ± SEM (*n* = 3; the significance of intergroup differences was estimated by one-way analysis of the variance (ANOVA), followed by Tukey’s multiple comparisons test).

To examine the protein level of BDNF and TrkB site-specific phosphorylation (pTyr^706/707^, pTyr^515^, pTyr^816^) in the different brain regions following UCMS and GSB-106 treatment a Western blotting was performed. Figure 5a shows that BDNF levels significantly decreased in the prefrontal cortex and the hippocampus (F = 18.5; *p =* 0.001 and F = 27.06; *p* = 0.001 respectively) of mice exposed to UCMS, compared to the control non-stressed animals, which is consistent with the previous data [54,55]. A chronic treatment with GSB-106 (1 mg/kg, per os, 26 days) during ongoing UCMS procedure restored UCMS-induced BDNF decrease in both brain structures (F_cortex_ = 18.5; *p* = 0.001 and F_hip_ = 27.06; *p* = 0.001) compared to that of the vehicle-treated UCMS mice. However, the administration of GSB-106 to animals that were not exposed to UCMS had no effect on BDNF content in these brain regions (F_cortex_ = 18.5; *p* = 0.98 and F_hip_ = 27.06; *p* = 0.07). 

Abundant evidence indicates that action mechanisms of pharmacologically diverse antidepressants implicate a phosphorylation and an activation/transactivation of the TrkB receptors in the brain regions is associated with the depression-like behavior [24]. Our next step was to perform immunoblotting with antibodies to specific phospho tyrosines of TrkB was performed in the prefrontal cortex and the hippocampus of chronically stressed mice treated with GSB-106. Figure 5b shows that 4-weeks of UCMS exposure did not alter Tyr^706/707^ phosphorylation in both structures (F_cortex_ = 8.26; *p* = 0.98; F_hip_ = 8.37; *p* = 0.89). Prolonged GSB-106 administration to UCMS subjected mice caused an increase in the pTrkB^706/707^ content in the prefrontal cortex (F = 8.26; *p* = 0.05) compared to UCMS animals, while the effect of GSB-106 on pTrkB^706/707^ level in both brain structures of non-stressed animals appeared to be statistically insignificant (F_cortex_ = 8.26; *p* = 0.65; F_hip_ = 8.37; *p* = 0.15).

As follows from the data presented in Figure 5c, a decrease in the pTrkB^816^ level was observed in the prefrontal cortex (F = 15.31; *p* = 0.0001) and in the hippocampus (F = 12.65; *p* = 0.0001) of UCMS mice compared to unstressed animals. Notably, data on TrkB^816^ phosphorylation in the prefrontal cortex of stressed animals shows bimodal distribution. However, none of the available data sets, other than those indicated, appear to be mathematically bimodal. At the same time, all our data pass the normality test (D’Agostino & Pearson normality test, Shapiro-Wilk normality test). Conceivably, the bimodal distribution of pTrkB^816^ values might reflect the individual variability in response to GSB-106 treatment in the cohort of participating animals.

Administration of GSB-106 for 26 days along with a continued stress procedure promoted a rise of TrkB phosphorylation at Tyr^816^ site in the prefrontal cortex (F = 15.31; *p* =0.0001) and in the hippocampus (F = 12.65; *p* = 0.0001) compared with control UCMS mice. Notably, in the absence of stress, GSB-106 caused a reduction of pTrkB^816^ in the prefrontal cortex (F = 15.31; *p* = 0.05), whereas in the hippocampus there was a non-significant trend for decreased TrkB phosphorylation at that tyrosine residue (F = 12.65; *p* = 0.09). We also found that UCMS produced a decrease in TrkB phosphorylation at Tyr^515^ site in the prefrontal cortex and the hippocampus (F = 8.92; *p* = 0.0006 and F = 4.4; *p* = 0.05, respectively) relative to control (Figure 5d). Further, we observed a decline of pTrkB^515^ in both structures (F_cortex_ = 8.92; *p* = 0.0015 and F_hip_ = 4.4; *p* = 0.045) of non-stressed mice after chronic GSB-106 treatment. However, no changes in pTrkB^515^ values were established in the prefrontal cortex and in the hippocampus of UCMS mice chronically treated with GSB-106 (F_cortex_ = 8.92; *p* = 0.059 and F_hip_ = 4.4; *p* = 0.11).

## 3. Discussion

The presented results for the first time show that prolonged per os GSB-106 administration (1 mg/kg, 26 days) produced an antidepressant-like behavioral response during an ongoing stress exposure in UCMS-treated Balb/c mice, as evaluated by the Porsolt test. Furthermore, GSB-106 restored a decrease in BDNF level induced by the UCMS procedure in the prefrontal cortex and in the hippocampus. Additionally, chronic administration of GSB-106 resulted in a TrkB activation (as estimated by phosphorylation of neurotrophin receptor at Tyr^Y706/707^ site) in the prefrontal cortex and induced a pronounced increase of TrkB^Y816^ phosphorylation in the prefrontal cortex and the hippocampus of UCMS subjected mice. Overall, these results indicate that GSB-106 exhibits an obvious antidepressant-like effect in a mouse model of UCMS-induced depression that was mediated, at least partially, by an increase in BDNF level and site-selective phosphorylation of TrkB^Y706/707^ and TrkB^Y816^. 

Two experimental design modifications were used to evaluate behavioral effects of GSB-106 on chronic stress. Initially, mice were exposed to stressful context for 42 days and 14 administrations of GSB-106 were carried out; then mice were subjected to a more prolonged stress procedure (54 days) and more GSB-106 treatments (26) were delivered. However, both experiments had one fundamental point in common, namely, they initially formed dysphoria-like chronic stress-induced behavior followed by treatment. Most studies utilizing this model of chronic stress have a similar design, where once the long-lasting stressful conditions are discontinued, recovery processes are likely to develop. In the present study, a 1 mg/kg dose of GSB-106 attenuated the depression-like responses of mice by 2.9 times following a 26-days treatment and by 1.2 times following a 14-days course, compared to the corresponding controls. 

It is well known that long-term exposure to stress, as well as other conditions that contribute to the depressive-like behavior in animals, is accompanied by a BDNF expression decrease in the prefrontal cortex and in the hippocampus, while an increase in the level of neurotrophin has been observed in the nucleus accumbens [56,57]. In particular, numerous studies reported a decrease in the BDNF mRNA or protein levels, as well as an attenuation of TrkB receptor activity (detected by the level of phosphorylation of tyrosine residues at Tyr^706/707^) and a decrease of Tyr^515^ and Tyr^816^ phosphorylation in the prefrontal cortex and hippocampus of rodents subjected to UCMS, that is consistent with the depression-like behavior [23,55,58]. In our study, a decrease of BDNF protein level both in the prefrontal cortex and the hippocampus in UCMS-induced depressed mice was found. Furthermore, a reduction of pTrkB^515^ and pTrkB^816^ content in the prefrontal cortex and the hippocampus of UCMS-treated mice was also revealed, while no changes in TrkB^706/707^ phosphorylation were observed in both brain structures of UCMS mice, generally indicating the alleviation of TrkB-dependent signaling in vulnerable brain regions of stressed animals. Overall, our behavioral and western-blot findings have demonstrated that UCMS produces depression-like symptoms (dysphoria), which are accompanied by the alterations of BDNF content and TrkB phosphorylation at specific tyrosine residues in the limbic brain structures, which are similar to those evidenced elsewhere. Importantly, the present study revealed that continuous UCMS procedure concomitantly with long-term per os GSB-106 treatment resulted in a BDNF content increase in the prefrontal cortex and the hippocampus and induction of site-specific phosphorylation of the TrkB receptor—at Tyr^706/707^ and Tyr^816^ sites, but not at Tyr^515^ site, in this way mimicking the pattern attributed for most of the conventional antidepressants. We consider that an increase in TrkB^706/707^ phosphorylation in the prefrontal cortex of stressed animals upon GSB-106 treatment might be a consequence of increased BDNF content, evoked by GSB-106, although it is quite difficult to determine the exact cause-and-effect relationships under the conditions of in vivo experiment with the multiple crosstalk and feedback loop mechanisms all of which suggest that this point requires additional investigations. Obtained data indicate the ability of GSB-106 to reverse depressive-like behavior. Simultaneously, GSB-106 normalizes the BDNF content, reduced due to a prolonged UCMS in animals, as well as the site-specific phosphorylation of TrkB receptor.

It has been experimentally established that the development of a depressive phenotype in UCMS is based on the elevated functionality of the hypothalamus-pituitary-adrenal (HPA) axis and its effects on the feedback systems of the limbic structures [59,60]. 

When a stressful event occurs, an activation of the HPA axis leads to the release of glucocorticoids and triggers a feedback mechanism, the main contributors of which are the hippocampus and the prefrontal cortex. As a result, the effects of stress are limited in time and the mechanism for restoring homeostasis and behavior is triggered. Prolonged activation of the HPA axis under chronic stress causes prolonged release of glucocorticoids which are toxic both for the hippocampus and the frontal cortex which leads to a decrease in the BDNF content [61,62], depletion of synaptic connections [63] and their morphological decrease develop [63,64].

In contrast to the hippocampus and the prefrontal cortex, the amygdala provides positive feedback to the HPA axis. Prolonged UCMS with the loss of hippocampal inhibition over the HPA axis increases activity in the amygdala. UCMS has been found to cause an increased coherence of activity in the amygdala [65], along with an increase in the dendrites’ length, the density of dendritic spines [66,67,68], and synaptic proteins [66]. The chronic stress activation causes a decrease in the activity of the mesolimbic dopamine system, which may lead to the development of anhedonia as a result of stress which could be a target of antidepressant effect [69].

It is widely believed that chronic antidepressant treatment causes an increase in the synthesis or release of BDNF, activation of TrkB receptors and related signal transduction pathways, triggers up-regulation of genes associated with BDNF-induced plasticity [7,24,70,71]. Duman and co-workers were the first, who have demonstrated the stimulating effect of chronic (21 days), but not acute tranylcypromine, sertraline, desipramine, mianserin treatment on the BDNF and TrkB mRNA expression in rat hippocampus [15]. Then, multiple studies supported the positive effect of prolonged antidepressant courses on the BDNF mRNA expression and their ability to prevent/abolish a decrease in BDNF protein level caused by a chronic stress paradigm in vivo [72,73,74,75,76,77].

Saarlainen et al. [18], Rantamaki et al. [19,20] have demonstrated that, unlike BDNF, which requires prolonged administration of antidepressants to get a level increase, activation of the TrkB receptor in the cortex and hippocampus can occur upon even acute drug treatment. Particularly, antidepressant-induced TrkB^706/707^ phosphorylation takes place within 30 min after a single dose, whereas no changes were detected at TrkB^515^ residue (Shc binding site) either under acute or chronic administration. Furthermore, an increase in Tyr^706/707^ phosphorylation was observed after sustained (21 days) antidepressant treatment as well [18]. However, phosphorylation at Tyr^816^ residue in the mice hippocampus was detected 1 h after acute administration of fluoxetine, riboxetine, citalopram, imipramine; a similar effect on the phosphorylation of Tyr^816^ was also established upon long-term administration (21 days) of fluoxetine [19]. Altogether, multiple complementary findings obtained to date, indicate that both acute and chronic courses of most clinically used antidepressants could induce the kinase activity of the receptor (increased phosphorylation at Tyr^706/707^), followed by an increase in Tyr^816^ (binding site for PLC-γ1). Notably, through preferential activation of TrkB-PLC-γ1-CREB-mediated signaling, antidepressants have been found to raise the level of synaptic plasticity in critical neural circuits to the degree required for relieving depressive symptoms, pointing out the significance of PLC-γ1/IP3/Ca^2+^ pathway for antidepressants response [78,79,80]. Our results on the tyrosine-specific phosphorylation of TrkB (Tyr^706/707^ and Tyr^816^ sites, but not at Tyr^515^) in the brain areas implicated in depression, caused by prolonged GSB-106 treatment in UCMS-exposed mice, are in line with the data observed on typical antidepressants, which provide TrkB-Tyr^816^/PLC-γ1 specific effects [18,19]. As was already mentioned, detailed animal studies revealed the substantial antidepressant-like activity of GSB-106 both in various tests (Porsolt, tail suspension, Nomura water wheel tests) and in animal depression models (chronic social defeat stress, LPS-induced anhedonia) [43,44,45,46]. Remarkably, it was clearly demonstrated that antidepressant-like properties of GSB-106 (acute, i.p.) depend on TrkB receptor activation and the PLC-γ1 signaling pathway in the Porsolt test in mice, as it was evident from the experiments with TrkB and PLC-γ1 inhibitors. GSB-106 (21 days, per os) has been shown to restore the reduced levels of synaptophysin and CREB in the hippocampus of C57BL/6 mice subjected to the 28-days chronic social defeat stress [45]. 

To our knowledge, this is the first report showing a site-selective phosphorylation of TrkB, elicited by small peptide or non-peptide BDNF analogs with antidepressant effects in the robust behavioral model of depression. Currently, several compounds with antidepressant properties were identified as putative ligands of the TrkB receptor, such as 7,8-dihydroxyflavone (TrkB agonist), ANA-12 (TrkB antagonist) and tetraterpenoid deoxygedunin. Zhang et al. demonstrated that chronic 7,8-dihydroxyflavone (28 days, i.p.) treatment induce antidepressant-like effect in mouse chronic mild stress model, accompanied by a restoration of BDNF level, TrkB phosphorylation at Tyr^706/707^ residue, a recovery of the synaptic proteins PSD95 and synaptophysin levels in the prefrontal cortex in CMS-subjected animals [33]. The antidepressant properties of 7,8-dihydroxyflavone and ANA-12 as well as their ability to increase BDNF and pTrkB-Tyr^706/707^ levels have also been demonstrated in a social defeat stress model and LPS-stimulated depressive-like behavior [81,82]. Another interesting fact is that 7,8-dihydroxyflavone treatment triggered phosphorylation of TrkB^Y816^ but not TrkB^Y515^ with subsequent activation of the PLC-γ1 pathway in primary striatal neurons and selectively reverted the decrease of pTyr^816^ but not pTyr^515^ in striatum of the chronically treated R6/1 transgenic mice [83]. 

Although the comprehensive evidence on the engagement of BDNF/TrkB signaling in antidepressant responses has been acquired, detailed mechanisms of this interaction remained unresolved until recently. Casarotto and colleagues reported the direct binding of both typical and rapid-acting antidepressants (in micromolar affinity) to the transmembrane region of TrkB dimers that promotes translocation and the synaptic localization of TrkB and facilitation of BDNF binding to TrkB and its activation. It is important to note that cholesterol has been shown to potentiate the effects of antidepressants and BDNF on TrkB signaling through the presumed impact on the conformation of TrkB dimers and the binding site of antidepressants. Furthermore, it was revealed that fluoxetine and ketamine increased Tyr^816^ phosphorylation in primary cortical neurons, whereas fluoxetine, imipramine, ketamine, *R,R*-HNK enhanced TrkB interaction with PLC-γ1 [22]. To date, ability to directly interact with the extracellular domain of TrkB and promote receptor dimerization was reported only for 7,8-dihydroxyflavone amongst the low-molecular weight putative ligands of TrkB with antidepressant properties [32]. Whilst GSB-106 has been shown to mediate its neurotrophic [40,84], neuroprotective [85,86] and antidepressant-like [87] effects through the TrkB-dependent pathways, the mechanisms, underlying this interplay remained rather elusive, since we have not yet explored the direct association of GSB-106 with TrkB. What is important, GSB-106 could produce an indirect receptor activation, implicated Src-dependent TrkB transactivation in SH-SY5Y serum-deprived cells [42], and elucidation of this issue in animal models will provide novel knowledge about the mechanisms of small TrkB ligands antidepressant action.

## 4. Materials and Methods

### 4.1. Animals

The experiments were conducted on 128 male Balb/c mice weighing 22–25 g (Research Center of Biomedical Technology, Federal Medical and Biological Agency, Russia). The animals were group-housed under standard conditions (9–12 mice per home cage), with a 12-h dark-light cycle at a temperature of 22 ± 2 °C and *ad libitum* access to granulated chow (MEST, Russia) and water. All experimental procedures were in compliance with the 322 the EC Directive 86/609/EEC for animal experiments and were approved by the bioethics committee of the FSBI “Research Zakusov Institute of Pharmacology” (protocol No. 1 of 31 January 2021). 

### 4.2. Substance

GSB-106 (MW 746.85; Figure 5) was synthesized as described previously [36] at Medicinal Chemistry Department of Zakusov Institute of Pharmacology.

### 4.3. Experimental Design

The UCMS model was used to induce chronic stress conditions [88]. While being maintained in a vivarium, animals were exposed to various stress factors, applied in a quasi-random manner (wet bedding, dirty boxes, water deprivation, reduction in daylight hours, etc.) within four weeks. Starting from day 29 stress factors application was accompanied by GSB-106, vehicle or amitriptyline treatment. The total duration of exposure to moderate chronic stress was either 42 or 54 days (Figure 1). The experimental design assumed the initiation of the treatment with the study drug after the manifestation of depressive-like responses in animals and its constant administration during the ongoing stress, which is usually applicable in similar experimental conditions and corresponds to the clinical situation. The in vivo experimental design was developed in compliance with the 3R principles. All compounds (GSB-106, amitriptyline) were dissolved in water immediately before administration. Compounds or vehicle were administered at a volume of 0.1 mL/10 g body weight.

The first series of behavioral experiments were focused on the ability of GSB-106 to reduce dysphoria under UCMS in comparison with the reference drug amitriptyline (Figure 3). Experimental animals were divided into five groups: (1) vehicle, no stress (*n* = 10); (2) vehicle, stress (*n* = 12); (3) GSB-106 (0.1 mg/kg), stress (*n* = 12); (4) GSB-106 (1 mg/kg), stress (*n* = 12); (5) amitriptyline (10 mg/kg), stress (*n* = 10). The experiment started with 28 days of UCMS exposure after which, in conjunction with continuing stress, the test substances were administered over the following 14 days (Figure 2a). Then, 60 min after the 14th treatment with vehicle, GSB-106 or amitriptyline, mice were subjected to the Porsolt test. 

The second experimental series consisted of two parallel stages—a behavioral assay and BDNF and pTrkB protein contents assessment (Figure 2b). Both stages began with the UCMS simulation for 28 days; on the 29th day of stress, the administration of GSB-106 or vehicle started (26 sequential per os treatments, 1 time per day) while stressing procedure was continued. 60 min after the 26th injection, 37 mice were subjected to Western-blot analysis. The following groups were formed: (1) vehicle, no stress (*n* = 9); (2) GSB-106 (1 mg/kg), no stress (*n* = 10); (3) vehicle, stress (*n* = 9); (4) GSB-106 (1 mg/kg), stress (*n* = 9). Behavioral tests were carried out with the animals of the second experimental cohort under a similar schedule for the control and the experimental groups. The Porsolt test was performed 60 min after 26th administration of the vehicle or GSB-106. 

### 4.4. Porsolt Test

The antidepressant activity was studied in the Porsolt test in mice [89]. Cylindrical transparent Plexiglas tanks (30 cm height × 10 cm diameter) were filled with water (25 ± 1 °C) up to 20 cm from the bottom. On the test day mice were put individually in the cylinders for a 6-min swim session, and from the 4th min it was video-taped marking the duration of immobility periods. Immobility was defined as a lack of activity other than that required from the animal to keep its head above water: tail movements and limited limb movements.

### 4.5. Western-Blotting

#### 4.5.1. Antibodies

Following primary antibodies were used in this study: rabbit polyclonal anti-BDNF (1:1000, #ab226843, Abcam, Cambridge, UK); rabbit monoclonal anti-TrkB (1:1000, #4603, Cell Signaling Technology, Danvers, MA, USA); anti-phospho-TrkB (Tyr^706/707^) (1:1000, #4621, Cell Signaling Technology, Danvers, MA, USA); rabbit polyclonal anti-phospho-TrkB (Tyr^816^) (1:1000, #NBP1-03499SS, Novus Biologicals, Centennial, CO, USA); anti-phospho-TrkB (Tyr^515^) (1:1000, # PA5-36695, Thermo Fisher Scientific, Waltham, MA, USA); mouse monoclonal anti-α-Tubulin (1:1000, #2125, Cell Signaling Technology, Danvers, MA, USA). Secondary antibodies conjugated to HRP (used at 1:12000 dilution) were from Cell Signaling Technology (Danvers, MA, USA) (anti-mouse IgG, #7076; anti-rabbit IgG, #7074).

#### 4.5.2. Samples Preparation

All mice were decapitated by cervical dislocation and the brain was rapidly removed. The hippocampus and prefrontal cortex were dissected on wet ice covered with filter paper dampened in 0.32 M sucrose solution at a temperature of 0–4 °C. Each brain structure was frozen in liquid nitrogen (−196 °C), weighed, and stored at −80 °C.

#### 4.5.3. Protein Preparation and Western Blot Analysis

Brain samples were homogenated and lysed with RIPA buffer (10^−2^ M Tris-Cl (pH 8.0), 10^−3^ M EDTA, 5 × 10^−4^ M EGTA, 1% Triton X-100, 0.1% sodium deoxycholate, 0.1% SDS, 0.14 × 10^−3^ M NaCl, 1 × protease inhibitor cocktail (#P8340), 1 × phosphatase inhibitor cocktail 2 and 3 (#P5726, P0044), 10^−3^ M PMSF (all from Sigma-Aldrich, St. Louis, MO, USA) using Tissue LyserLT (Quiagen, Hombrechtikon, Switzerland). Protein concentration was determined using BCA Protein Assay Kit (#23225, Thermo Fisher Scientific, Waltham, MA, USA). Lysates were loaded on SDS-PAGE, and separated proteins were transferred onto the nitrocellulose membranes (#GE10600002, Sigma-Aldrich, St. Louis, MO, USA). The membranes were blocked in 5% not-fat milk or 5% BSA in TBS-T for 60 min at room temperature, incubated with the appropriate primary antibody (+4 °C, overnight) and then with a secondary antibody conjugated with HRP (60 min at room temperature). Bands were visualized using SignalFire Plus ECL Reagent (#12630, Cell Signaling Technology, Danvers, MA, USA). Membranes were scanned using Amersham Imager 680 (GE HealthCare, Chicago, IL, USA) and quantified in the Image Quant TL v.8.1 (GE HealthCare, Chicago, IL, USA). Measurement of each protein was then normalized on the related α-Tubulin loading control.

### 4.6. Statistical Analysis

Analysis was performed using the GraphPad Prizm 7.0 software (GraphPad, La Jolla, CA, USA, www.graphpad.com). The normal distribution of the data was evaluated using the Shapiro–Wilk test. The results are presented as means ± SEM. The significance of intergroup differences was estimated by one-way analysis of the variance (ANOVA), followed by Tukey’s multiple comparisons test.

## 5. Conclusions

Altogether, irrespective of the mode of GSB-106 interaction with the TrkB, prolonged per os treatment with dimeric dipeptide ameliorates depressive-like behavior in UCMS-stressed mice. Remarkably, our findings strongly suggest that an increase in BDNF in the prefrontal cortex and hippocampus, as well as the restoration of phosphorylation at the TrkB^Y706/707^ (prefrontal cortex) and at the PLC-γ1 interaction site TrkB^Y816^ (prefrontal cortex and hippocampus) of UCMS mice might contribute to antidepressant-like mechanisms of GSB-106.

## Data Availability

The datasets generated during the current study are available from the corresponding authors on reasonable request.

## Abbreviations

Akt	AKT serine/threonine kinase
ANA-12	*N*-[2-[[(Hexahydro-2-oxo-1H-azepin-3-yl)amino]carbonyl]phenyl]-benzo[b]thiophene-2-carboxamide
BAD	BCL-2 associated agonist of cell death
BDNF	Brain-derived neurotrophic factor
BSA	Bovine serum albumin
CMS	Chronic mild stress
CREB	Cyclic AMP-responsive element-binding protein
EDTA	Ethylenediaminetetraacetic acid
EGTA	Ethylene glycol-bis(2-aminoethylether)-*N*,*N*,*N*′,*N*′-tetraacetic acid
Erk	Mitogen-activated protein kinase; extracellular-signal-regulated kinase
HRP	Horseradish peroxidase
IP3	Inositol 1,4,5-trisphosphate
LPS	Lipopolysaccharide
MAPK	Mitogen-activated protein kinase
mTORC1	Mammalian/mechanistic target of rapamycin complex 1
NMDA	(2R)-2-(methylamino)butanedioic acid
PI3K	Phosphatidylinositol 3-kinase
PLCγ	1-phosphatidylinositol 4,5-bisphosphate phosphodiesterase gamma
PMSF	Phenylmethylsulfonyl fluoride
PSD95	Postsynaptic density protein 95
*R,R*-HNK	(*2R,6R*)-hydroxynorketamine
SDS-PAGE	Sodium dodecyl sulfate polyacrylamide gel electrophoresis
TBS-T	Tris-buffered saline with 0.1% Tween
TrkB	BDNF/NT-3 growth factors receptor; neurotrophic receptor tyrosine kinase 2
UCMS	Unpredictable chronic mild stress

## References

[1] Institute of Health Metrics and Evaluation Global Health Data Exchange (GHDx). http://ghdx.healthdata.org/gbd-results-tool?params=gbd-api-2019-permalink/d780dffbe8a381b25e1416884959e88b.

[2] Pekala K., Budzynska B., Biala G. (2014). Utility of the chronic unpredictable mild stress model in research on new antidepressants. Curr. Issues Pharm. Med. Sci..

[3] Krishnan V., Nestler E.J. (2008). The molecular neurobiology of depression. Nature.

[4] Campbell S., Marriott M., Nahmias C., MacQueen G.M. (2004). Lower hippocampal volume in patients suffering from depression: A meta-analysis. Am. J. Psychiatry..

[5] Lucassen P.J., Muller M.B., Holsboer F., Bauer J., Holtrop A., Wouda J., Hoogendijk W.J., De Kloet E.R., Swaab D.F. (2001). Hippocampal apoptosis in major depression is a minor event and absent from subareas at risk for glucocorticoid overexposure. Am. J. Pathol..

[6] Nestler E.J., Barrot M., DiLeone R.J., Eisch A.J., Gold S.J., Monteggia L.M. (2002). Neurobiology of depression. Neuron.

[7] Duman R.S., Monteggia L.M. (2006). A neurotrophic model for stress-related mood disorders. Biol. Psychiatry.

[8] Duman R.S., Aghajanian G.K. (2012). Synaptic dysfunction in depression: Potential therapeutic targets. Science.

[9] Ménard C., Hodes G.E., Russo S.J. (2016). Pathogenesis of depression: Insights from human and rodent studies. Neuroscience.

[10] Spellman T., Liston C. (2020). Toward Circuit Mechanisms of Pathophysiology in Depression. Am. J. Psychiatry.

[11] Alvano S.A., Zieher L.M. (2020). An updated classification of antidepressants: A proposal to simplify treatment. Per. Med. Psychiatry.

[12] Rush A.J., Trivedi M.H., Wisniewski S.R., Nierenberg A.A., Stewart J.W., Warden D., Niederehe G., Thase M.E., Lavori P.W., Lebowitz B.D. (2006). Acute and longer-term outcomes in depressed outpatients requiring one or several treatment steps: A STAR*D report. Am. J. Psychiatry.

[13] Duman R.S., Aghajanian G.K., Sanacora G., Krystal J.H. (2016). Synaptic plasticity and depression: New insights from stress and rapid-acting antidepressants. Nat. Med..

[14] Covington H.E., Vialou V., Nestler E.J. (2010). From synapse to nucleus: Novel targets for treating depression. Neuropharmacology.

[15] Nibuya M., Morinobu S., Duman R.S. (1995). Regulation of BDNF and trkB mRNA in rat brain by chronic electroconvulsive seizure and antidepressant drug treatments. J. Neurosci..

[16] Castrén E., Rantamäki T. (2010). The role of BDNF and its receptors in depression and antidepressant drug action: Reactivation of developmental plasticity. Dev. Neurobiol..

[17] Pittenger C., Duman R. (2008). Stress, depression, and neuroplasticity: A convergence of mechanisms. Neuropsychopharmacology.

[18] Saarelainen T., Hendolin P., Lucas G., Koponen E., Sairanen M., MacDonald E., Agerman K., Haapasalo A., Nawa H., Aloyz R. (2003). Activation of the TrkB neurotrophin receptor is induced by antidepressant drugs and is required for antidepressant-induced behavioral effects. J. Neurosci..

[19] Rantamäki T., Hendolin P., Kankaanpää A., Mijatovic J., Piepponen P., Domenici E., Chao M.V., Männistö P.T., Castrén E. (2007). Pharmacologically diverse antidepressants rapidly activate brain-derived neurotrophic factor receptor TrkB and induce phospholipase-Cgamma signaling pathways in mouse brain. Neuropsychopharmacology.

[20] Rantamäki T., Vesa L., Antila H., Di Lieto A., Tammela P., Schmitt A., Lesch K.P., Rios M., Castrén E. (2011). Antidepressant drugs transactivate TrkB neurotrophin receptors in the adult rodent brain independently of BDNF and monoamine transporter blockade. PLoS ONE.

[21] Gould T.D., Zarate C.A., Thompson S.M. (2019). Molecular pharmacology and neurobiology of rapid-acting antidepressants. Annu. Rev. Pharmacol. Toxicol..

[22] Casarotto P.C., Girych M., Fred S.M., Kovaleva V., Moliner R., Enkavi G., Biojone C., Cannarozzo C., Sahu M.P., Kaurinkoski K. (2021). Antidepressant drugs act by directly binding to TRKB neurotrophin receptors. Cell.

[23] Rantamäki T., Castrén E. (2008). Targeting TrkB neurotrophin receptor to treat depression. Expert Opin. Ther. Targets.

[24] Rantamäki T. (2019). TrkB neurotrophin receptor at the core of antidepressant effects, but how?. Cell Tissue Res..

[25] Castrén E., Monteggia L.M. (2021). Brain-derived neurotrophic factor signaling in depression and antidepressant action. Biol. Psychiatry.

[26] Autry A.E., Monteggia L.M. (2012). Brain-derived neurotrophic factor and neuropsychiatric disorders. Pharmacol. Rev..

[27] Longo F.M., Massa S.M. (2013). Small-molecule modulation of neurotrophin receptors: A strategy for the treatment of neurological disease. Nat. Rev. Drug Discov..

[28] Kashyap M.P., Roberts C., Waseem M., Tyagi P. (2018). Drug targets in neurotrophin signaling in the central and peripheral nervous system. Mol. Neurobiol..

[29] Josephy-Hernandez S., Jmaeff S., Pirvulescu I., Aboulkassim T., Saragovi H.U. (2017). Neurotrophin receptor agonists and antagonists as therapeutic agents: An evolving paradigm. Neurobiol. Dis..

[30] Siuciak J.A., Lewis D.R., Wiegand S.J., Lindsay R.M. (1997). Antidepressant-like effect of brain-derived neurotrophic factor (BDNF). Pharmacol. Biochem. Behav..

[31] Shirayama Y., Chen A.C., Nakagawa S., Russell D.S., Duman R.S. (2002). Brain-derived neurotrophic factor produces antidepressant effects in behavioral models of depression. J. Neurosci..

[32] Jang S.-W., Liu X., Yepes M., Shepherd K.R., Miller G.W., Liu Y., Wilson W.D., Xiao G., Blanchi B., Sun Y.E. (2010). A selective TrkB agonist with potent neurotrophic activities by 7,8-dihydroxyflavone. Proc. Natl. Acad. Sci. USA.

[33] Zhang M.-W., Zhang S.-F., Li Z.-H., Han F. (2016). 7,8-Dihydroxyflavone reverses the depressive symptoms in mouse chronic mild stress. Neurosci. Lett..

[34] Liu X., Chan C.-B., Jang S.-W., Pradoldej S., Huang J., He K., Phun L.H., France S., Xiao G., Jia Y. (2010). A synthetic 7,8-dihydroxyflavone derivative promotes neurogenesis and exhibits potent antidepressant effect. J. Med. Chem..

[35] Cazorla M., Prémont J., Mann A., Girard N., Kellendonk C., Rognan D. (2011). Identification of a low-molecular weight TrkB antagonist with anxiolytic and antidepressant activity in mice. J. Clin. Investig..

[36] Gudasheva T.A., Tarasiuk A.V., Pomogaibo S.V., Logvinov I.O., Povarnina P.I., Antipova T.A., Seredenin S.B. (2012). Design and synthesis of dipeptide mimetics of the brain-derived neurotrophic factor. Russ. J. Bioorg. Chem..

[37] Ibáñez C.F. (1995). Neurotrophic factors: From structure-function studies to designing effective therapeutics. Trends Biotechnol..

[38] Pattarawarapan M., Burgess K. (2003). Molecular basis of neurotrophin-receptor interactions. J. Med. Chem..

[39] FSBI Zakusov Institute of Pharmacology (2011). Dipeptide Mimetics of NGF and BDNF Neurotrophins. RU Patent.

[40] Gudasheva T.A., Logvinov I.O., Antipova T.A., Seredenin S.B. (2013). Brainderived neurotrophic factor loop 4 dipeptide mimetic GSB-106 activates TrkB, Erk, and Akt and promotes neuronal survival in vitro. Dokl. Biochem. Biophys..

[41] Gudasheva T.A., Logvinov I.O., Nikolaev S.V., Antipova T.A., Povarnina P.Y., Seredenin S.B. (2020). Dipeptide mimetics of different NGF and BDNF loops activate PLC-γ1. Dokl. Biochem. Biophys..

[42] Zainullina L.F., Vakhitova Y.V., Lusta A.Y., Gudasheva T.A., Seredenin S.B. (2021). Dimeric mimetic of BDNF loop 4 promotes survival of serum-deprived cell through TrkB-dependent apoptosis suppression. Sci. Rep..

[43] Seredenin S.B., Voronina T.A., Gudasheva T.A., Garibova T.L., Molodavkin G.M., Litvinova S.A., Elizarova E.A., Poseva V.I. (2013). Antidepressant effect of dimeric dipeptide GSB-106, an original low-molecular-weight mimetic of BDNF. Acta Nat..

[44] Povarnina P.Y., Garibova T.L., Gudasheva T.A., Seredenin S.B. (2018). Antidepressant effect of an orally administered dipeptide mimetic of the brain-derived neurotrophic factor. Acta Nat..

[45] Gudasheva T.A., Tallerova A.V., Mezhlumyan A.G., Antipova T.A., Logvinov I.O., Firsova Y.N., Povarnina P.Y., Seredenin S.B. (2021). Low-molecular weight BDNF mimetic, dimeric dipeptide GSB-106, reverses depressive symptoms in mouse chronic social defeat stress. Biomolecules.

[46] Tallerova A.V., Mezhlumyan A.G., Yarkova M.A., Gudasheva T.A., Seredenin S.B. (2021). Effects of Original Compounds GSB-106, GML-3, and GZK-111 in an experimental lipopolysaccharide-induced anhedonia model. Pharm. Chem. J..

[47] Willner P. (2017). The chronic mild stress (CMS) model of depression: History, evaluation and usage. Neurobiol. Stress..

[48] Porsolt R.D. (2000). Animal models of depression: Utility for transgenic research. Rev. Neurosci..

[49] Willner P. (1997). Validity, reliability and utility of the chronic mild stress model of depression: A 10-year review and evaluation. Psychopharmacology.

[50] Tornese P., Sala N., Bonini D., Bonifacino T., La Via L., Milanese M., Treccani G., Seguini M., Ieraci A., Mingardi J. (2019). Chronic mild stress induces anhedonic behavior and changes in glutamate release, BDNF trafficking and dendrite morphology only in stress vulnerable rats. The rapid restorative action of ketamine. Neurobiol. Stress.

[51] Kudryashov N.V., Kalinina T.S., Shimshirt A.A., Narkevich V.B., Naplekova P.L., Kasabov K.A., Kudrin V.S., Voronina T.A., Fisenko V.P. (2020). The behavioral and neurochemical aspects of the interaction between antidepressants and unpredictable chronic mild stress. Acta Nat..

[52] Belzeaux R., Gorgievski V., Fiori L.M., Lopez J.P., Grenier J., Lin R., Nagy C., Ibrahim E.C., Gascon E., Courtet P. (2020). GPR56/ADGRG1 is associated with response to antidepressant treatment. Nature Commun..

[53] Yalcin I., Belzung C., Surget A. (2008). Mouse strain differences in the unpredictable chronic mild stress: A four-antidepressant survey. Behav. Brain Res..

[54] Larsen M.H., Mikkelsen J.D., Hay-Schmidt A., Sandi C. (2010). Regulation of brain-derived neurotrophic factor (BDNF) in the chronic unpredictable stress rat model and the effects of chronic antidepressant treatment. J. Psychiatr. Res..

[55] Hill M.N., Hellemans K.G.C., Verma P., Gorzalka B.B., Weinberg J. (2012). Neurobiology of chronic mild stress: Parallels to major depression. Neurosci. Biobehav. Rev..

[56] Yang C., Shirayama Y., Zhang J.-C., Ren Q., Hashimoto K. (2015). Regional differences in brain-derived neurotrophic factor levels and dendritic spine density confer resilience to inescapable stress. Int. J. Neuropsychopharmacol..

[57] Qiao H., An S.-C., Xu C., Ma X.-M. (2017). Role of proBDNF and BDNF in dendritic spine plasticity and depressive-like behaviors induced by an animal model of depression. Brain Res..

[58] Li X., Wang H., Chen Q., Li Z., Liu C., Yin S., You Z. (2019). Felbamate produces antidepressant-like actions in the chronic unpredictable mild stress and chronic social defeat stress models of depression. Fundam. Clin. Pharmacol..

[59] Mark D., Kvarta M.D., Bradbrook K.E., Dantrassy H.M., Bailey A.M., Thompson S.M. (2015). Corticosterone mediates the synaptic and behavioral effects of chronic stress at rat hippocampal temporoammonic synapses. J. Neurophysiol..

[60] Chen J., Wang Z.-Z., Zuo W., Zhang S., Chu S.-F., Chen N.-H. (2016). Effects of chronic mild stress on behavioral and neurobiological parameters—Role of glucocorticoid. Horm. Behav..

[61] Grønli J., Bramham C., Murison R., Kanhema T., Fiske E., Bjorvatn B., Ursin R., Portas C.M. (2006). Chronic mild stress inhibits BDNF protein expression and CREB activation in the dentate gyrus but not in the hippocampus proper. Pharmacol. Biochem. Behav..

[62] Filho C.B., Jesse C.R., Donato F., Giacomeli R., Del Fabbro L., da Silva Antunes M., de Gomes M.G., Goes A.T.R., Boeira S.P., Prigol M. (2015). Chronic unpredictable mild stress decreases BDNF and NGF levels and Na^+^,K^+^-ATPase activity in the hippocampus and prefrontal cortex of mice: Antidepressant effect of chrysin. Neuroscience.

[63] Bessa J., Ferreira D., Melo I., Marques F., Cerqueira J.J., Palha J.A., O F X Almeida O.F.X., Sousa N. (2009). The mood-improving actions of antidepressants do not depend on neurogenesis but are associated with neuronal remodeling. Mol. Psychiatry.

[64] Delgado y Palacios R., Campoa A., Henningsenc K., Verhoyea M., Pootb D., Dijkstrad J., Van Audekerkea J., Benvenistee H., Sijbersb J., Wiborgc O. (2011). Magnetic resonance imaging and spectroscopy reveal differential hippocampal changes in anhedonic and resilient subtypes of the chronic mild stress rat model. Magnetic resonance imaging and spectroscopy reveal differential hippocampal changes in anhedonic and resilient subtypes of the chronic mild stress rat model. Biol. Psychiatry.

[65] Delgado y Palacios R., Verhoye M., Henningsen K., Wiborg O., Van der Linden A. (2014). Diffusion kurtosis imaging and high-resolution MRI demonstrate structural aberrations of caudate putamen and amygdala after chronic mild stress. PLoS ONE.

[66] Li X.-L., Yuan Y.-G., Xu H., Wu D., Gong W.-G., Geng L.-Y., Wu F.-F., Tang H., Xu L., Zhang Z.-J. (2015). Changed synaptic plasticity in neural circuits of depressive-like and escitalopram-treated rats. Int. J. Neuropsychopharmacol..

[67] Sharma H.R., Thakur M.K. (2015). Correlation of ERα/ERβ expression with dendritic and behavioural changes in CUMS mice. Physiol. Behav..

[68] Qiao H., Li M.-X., Xu C., Chen H.-B., An S.-C., Ma X.-M. (2016). Dendritic spines in depression: What we learned from animal models. Neural. Plast..

[69] Di Chiara G., Loddo P., Tanda G. (1999). Reciprocal changes in prefrontal and limbic dopamine responsiveness to aversive and rewarding stimuli after chronic mild stress: Implications for the psychobiology of depression. Biol. Psychiatry..

[70] Castrén E. (2004). Neurotrophic effects of antidepressant drugs. Curr. Opin. Pharmacol..

[71] Duman R.S., Voleti B. (2012). Signaling pathways underlying the pathophysiology and treatment of depression: Novel mechanisms for rapid-acting agents. Trends Neurosci..

[72] Dias B.G., Banerjee S.B., Duman R.S., Vaidya V.A. (2003). Differential regulation of brain derived neurotrophic factor transcripts by antidepressant treatments in the adult rat brain. Neuropharmacology.

[73] Russo-Neustadt A., Beard R.C., Cotman C.W. (1999). Exercise, antidepressant medications, and enhanced brain derived neurotrophic factor expression. Neuropsychopharmacology.

[74] Tsankova N.M., Berton O., Renthal W., Kumar A., Neve R.L., Nestler E.J. (2006). Sustained hippocampal chromatin regulation in a mouse model of depression and antidepressant action. Nat. Neurosci..

[75] Alme M.N., Wibrand K., Dagestad G., Bramham C.R. (2007). Chronic fluoxetine treatment induces brain region-specific upregulation of genes associated with BDNF-induced long-term potentiation. Neural. Plast..

[76] Russo-Neustadt A., Ha T., Ramirez R., Kesslak J.P. (2001). Physical activity-antidepressant treatment combination: Impact on brain-derived neurotrophic factor and behavior in an animal model. Behav. Brain Res..

[77] Altar C.A., Whitehead R.E., Chen R., Wortwein G., Madsen T.M. (2003). Effects of electroconvulsive seizures and antidepressant drugs on brain-derived neurotrophic factor protein in rat brain. Biol. Psychiatry.

[78] Minichiello L., Calella A.M., Medina D.L., Bonhoeffer T., Klein R., Korte M. (2002). Mechanism of TrkB-mediated hippocampal long-term potentiation. Neuron.

[79] Yagasaki Y., Numakawa T., Kumamaru E., Hayashi T., Su T.-P., Kunugi H. (2006). Chronic antidepressants potentiate via sigma-1 receptors the brain-derived neurotrophic factor-induced signaling for glutamate release. J. Biol. Chem..

[80] Koponen E., Võikar V., Riekki R., Saarelainen T., Rauramaa T., Rauvala H., Taira T., Castrén E. (2004). Transgenic mice overexpressing the full-length neurotrophin receptor trkB exhibit increased activation of the trkB-PLCgamma pathway, reduced anxiety, and facilitated learning. Mol. Cell. Neurosci..

[81] Zhang J.-C., Yao W., Dong C., Yang C., Ren Q., Ma M., Han M., Hashimoto K. (2015). Comparison of ketamine, 7,8-dihydroxyflavone, and ANA-12 antidepressant effects in the social defeat stress model of depression. Psychopharmacology.

[82] Zhang J.-C., Wu J., Fujita Y., Yao W., Ren Q., Yang C., Li S.-X., Shirayama Y., Hashimoto K. (2014). Antidepressant effects of TrkB ligands on depression-like behavior and dendritic changes in mice after inflammation. Int. J. Neuropsychopharmacol..

[83] García-Díaz Barriga G., Giralt A., Anglada-Huguet M., Gaja-Capdevila N., Orlandi J.G., Soriano J., Canals J.-M., Alberch J. (2017). 7,8-dihydroxyflavone ameliorates cognitive and motor deficits in a Huntingtonn’s disease mouse model through specific activation of the PLCγ1 pathway. Hum. Mol. Genet..

[84] Zainullina L.F., Gudasheva T.A., Vakhitova Y.V., Seredenin S.B. (2019). Low-molecular-weight compound GSB-106 mimics the cellular effects of BDNF after serum deprivation. Dokl. Biochem. Biophys..

[85] Gudasheva T.A., Povarnina P.Y., Logvinov I.O., Antipova T.A., Seredenin S.B. (2016). Mimetics of brain-derived neurotrophic factor loops 1 and 4 are active in a model of ischemic stroke in rats. Drug Des. Devel. Ther..

[86] Povarnina P.Y., Gudasheva T.A., Seredenin S.B. (2018). Dimeric dipeptide mimetics of NGF and BDNF are promising agents for post-stroke therapy. J. Biomed. Sci. Eng..

[87] Gudasheva T.A., Povarnina P.Y., Tarasiuk A.V., Seredenin S.B. (2019). The low molecular weight Brain-derived neurotrophic factor mimetics with antidepressant-like activity. Curr. Pharm. Des..

[88] Grønli J., Murison R., Fiske E., Bjorvatn B., Sørensen E., Portas C.M., Ursin R. (2005). Effects of chronic mild stress on sexual behavior, locomotor activity and consumption of sucrose and saccharine solutions. Physiol. Behav..

[89] Porsolt R.D., Bertin A., Jalfre M. (1978). “Behavioural despair” in rats and mice: Strain differences and the effects of imipramine. Eur. J. Pharmacol..

